# Effectiveness of CHIKV vaccine VLA1553 demonstrated by passive transfer of human sera

**DOI:** 10.1172/jci.insight.160173

**Published:** 2022-07-22

**Authors:** Pierre Roques, Andrea Fritzer, Nathalie Dereuddre-Bosquet, Nina Wressnigg, Romana Hochreiter, Laetitia Bossevot, Quentin Pascal, Fabienne Guehenneux, Annegret Bitzer, Irena Corbic Ramljak, Roger Le Grand, Urban Lundberg, Andreas Meinke

**Affiliations:** 1 Université Paris-Saclay, INSERM, CEA, Center for Immunology of Viral, Auto-Immune, Hematological and Bacterial diseases (IMVA-HB/IDMIT), Fontenay-aux-Roses, France.; 2 Valneva Austria GmbH, Campus Vienna Biocenter 3, Vienna, Austria.; 3 Valneva SE, Saint Herblain, France.

**Keywords:** Infectious disease, Vaccines, Immunoglobulins, Immunotherapy

## Abstract

Chikungunya virus (CHIKV) is a reemerging mosquito-borne alphavirus responsible for numerous outbreaks. Chikungunya can cause debilitating acute and chronic disease. Thus, the development of a safe and effective CHIKV vaccine is an urgent global health priority. This study evaluated the effectiveness of the live-attenuated CHIKV vaccine VLA1553 against WT CHIKV infection by using passive transfer of sera from vaccinated volunteers to nonhuman primates (NHP) subsequently exposed to WT CHIKV and established a serological surrogate of protection. We demonstrated that human VLA1553 sera transferred to NHPs conferred complete protection from CHIKV viremia and fever after challenge with homologous WT CHIKV. In addition, serum transfer protected animals from other CHIKV-associated clinical symptoms and from CHIKV persistence in tissue. Based on this passive transfer study, a 50% micro–plaque reduction neutralization test titer of ≥ 150 was determined as a surrogate of protection, which was supported by analysis of samples from a seroepidemiological study. In conclusion, considering the unfeasibility of an efficacy trial due to the unpredictability and explosive, rapidly moving nature of chikungunya outbreaks, the definition of a surrogate of protection for VLA1553 is an important step toward vaccine licensure to reduce the medical burden caused by chikungunya.

## Introduction

The vector-borne chikungunya virus (CHIKV) disease is caused by CHIKV, a member of the alphavirus family. The disease is extremely difficult to eradicate because the virus is maintained in nature by propagation among arthropod vectors and their hosts, without the need of human-to-human contact for transmission (1, 2). Since its reemergence in 2005 in the West Indian Ocean region, CHIKV spread close to all areas in the world where its main vectors *Aedes aegypti* and *Ae*. *albopictus* mosquitoes can be found. After 2005, multiple outbreaks and large epidemics of chikungunya (CHIK) emerged across different continents including Asia, Europe, and North America (3–5). Specifically, South America and Southeast Asia were affected by outbreaks in recent years (6, 7). CHIKV infections are characterized by acute febrile disease accompanied by headache, muscle pain, and skin rash, which results in chronic and incapacitating arthralgia in up to 60% of patients (8–10). Furthermore, patients may suffer from severe and often debilitating joint pain, which can persist for years, especially in adults (11, 12). There is, therefore, an urgent demand for effective prophylaxis.

Several promising vaccine candidates against CHIKV are currently in clinical development (13, 14). The most advanced prophylactic vaccine against CHIKV to date is the live-attenuated vaccine VLA1553, which was initially studied in a common European effort (ICRES FP7-HEALTH project; https://cordis.europa.eu/project/id/261202) and then further developed by Valneva. The vaccine was tested in both mouse and nonhuman primate (NHP) models, as well as in a phase I clinical trial (15–17). In NHPs, a single vaccination was demonstrated to be safe and protected all animals from WT CHIKV challenge using strain LR-2006-OPY1 (La Reunion strain of East Central South African genotype) with more than 100 times the 50% animal infectious dose (AID_50_) (16).

Subsequently, VLA1553 was subject to a phase I dose-escalation study (NCT03382964; https://clinicaltrials.gov/ct2/show/NCT03382964) involving 120 healthy volunteers from 18 to 45 years of age (17). This study showed a very good safety and immunogenicity profile with a seroconversion rate of 100% that was sustained for at least 1 year after a single immunization. A single vaccination was sufficient to induce high titer neutralizing antibodies, as shown by the absence of an anamnestic response in more than 96% of all participants after revaccination. In addition, vaccinees were protected from VLA1553-induced viremia after revaccination.

For CHIK, it is widely accepted that immunity against CHIKV infection and disease is conferred by neutralizing antibodies (18). Preclinical studies in mice and NHPs provided evidence that antibodies play an important protective role against acute CHIKV infection. Indeed, B cell–deficient mice were unable to clear CHIKV viremia, contrary to WT mice (19). Specifically, passive transfer of CHIKV-specific immune sera conferred protection against disease to recipient mice, whereas adoptive transfer of primed CD8^+^ T cells had no impact on viremia (20–23). Furthermore, a combination of neutralizing monoclonal antibodies protected against a lethal CHIKV challenge in a mouse model (24) and the application of human neutralizing monoclonal antibodies blocked CHIKV spread and inflammation in NHPs (25). Studies with several vaccine candidates demonstrated that vaccines inducing neutralizing antibodies protected against infection, whereas vaccines inducing mainly CD8^+^ T cells did not protect (26). Importantly, preclinical results are strongly supported by findings from natural CHIKV infections in humans. Robust IgM/IgG neutralizing antibody responses that primarily target E1/E2 structural proteins are elicited following CHIKV infection in humans. In addition, natural CHIKV infection induces a durable neutralizing antibody response that is believed to confer life-long immunity (27, 28).

Acknowledging that late-stage clinical development has been hampered by the unpredictability of CHIKV outbreaks, barriers to traditional vaccine development and licensure require novel strategies such as the definition of a surrogate of protection (29). The important role of neutralizing antibodies for protection provided the basis to perform a passive transfer study in NHPs using human sera from the VLA1553 phase I study. Ideally, the definition of a surrogate should also be supported by seroepidemiology data, as both approaches individually have their strengths and limitations. Thus, the aims of this study were (a) to provide evidence that VLA1553-induced antibodies in human sera can provide protection in a NHP challenge model and (b) to establish a serological surrogate of protection for VLA1553 based on both data derived from the NHP passive transfer study and on seroepidemiological data.

Here, the correlation between antibody titers of serum samples from VLA1553 phase I vaccinees and the protection of NHPs from WT CHIKV-induced viremia and fever upon transfer of VLA1553 phase I serum pools was analyzed. Using VLA1553 phase I serum pools with various titers, a threshold neutralizing antibody titer for protection after vaccination with VLA1553 was established at a micro–plaque reduction neutralization test 50% (μPRNT_50_) titer of ≥ 150. To further support the surrogate of protection determined in the NHP passive transfer study, a panel of sera from a prospective longitudinal seroepidemiological cohort study from the Philippines (30) was evaluated in the same neutralization assay. Yoon and colleagues (30) had proposed that a PRNT_80_ titer ≥ 10 correlated with protection from symptomatic CHIKV infection, as observed in their seroepidemiological study. The translation of the proposed PRNT_80_ titer ≥ 10 into a μPRNT_50_ titer in our neutralization assay was in agreement with the surrogate of protection established in the NHP passive transfer study using VLA1553 serum samples from the phase I clinical trial.

## Results

### Study design.

**Figure 1:**
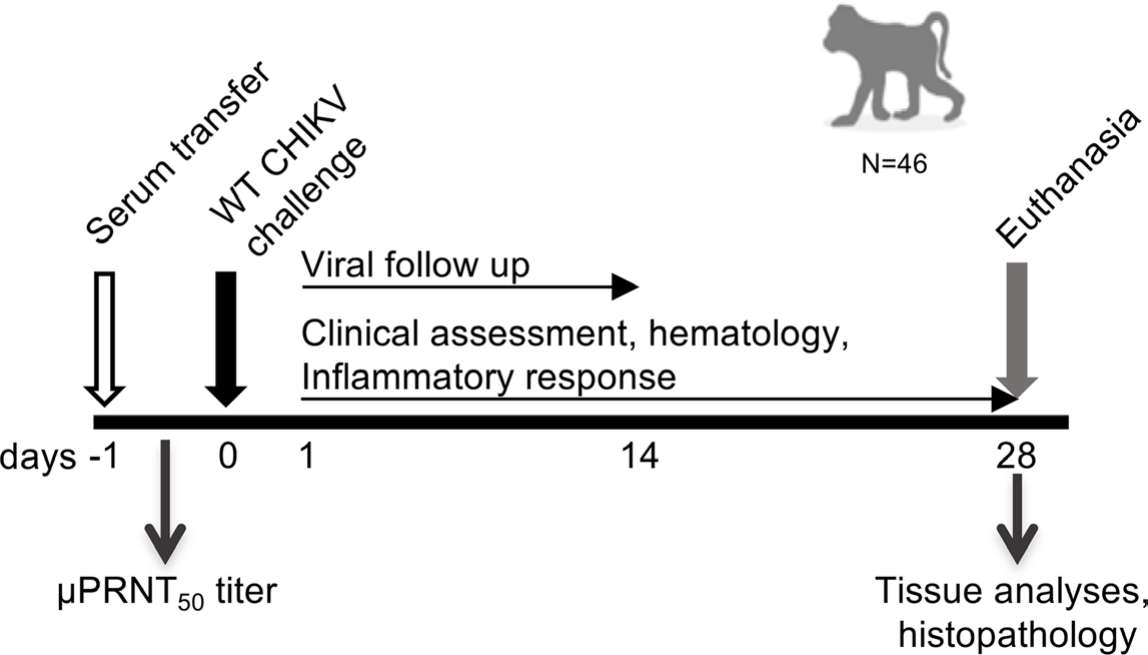
Study design. VLA1553 phase I serum transfer was performed on d–1. Just prior to WT CHIKV challenge on d0, a plasma sample was drawn from each animal to determine the μPRNT_50_ titer after serum transfer. Plasma viremia was determined from d1 up to d14 after challenge. Clinical assessments including daily temperature measurements and full hematology, as well as analyses of cytokine/chemokine profiles, were conducted up to d28 after challenge. On d28 after challenge, NHPs were euthanized and tissue samples were collected for analysis of the presence of CHIKV.

The aim of the study was to assess the effectiveness of the live-attenuated CHIKV vaccine VLA1553 by passive transfer of VLA1553-101 serum pools from vaccinated volunteers and to demonstrate the correlation between antibody titers and the protection of cynomolgus macaques (*Macaca fascicularis*) from WT CHIKV-induced viremia and fever. For this purpose, as shown in Figure 1, human nonimmune serum (controls) and human VLA1553 phase I serum pools were transferred i.v. on day –1 (d–1) to NHPs. Human serum pools obtained from various time-points after vaccination (d14, d28, d84, and — to assess persistence of the immune response — from d180) had various micro neutralization test (μNT_50_) titers ranging from ultralow titer serum (ULS), low titer serum (LS), medium titer serum (MS), and medium high titer serum (MHS) to high titer serum (HS) (Table 1). One day after human serum transfer (d0), just prior to challenge with WT CHIKV LR2006-OPY1, a blood sample was drawn from each animal to measure neutralizing anti-CHIKV antibody titers by the same μPRNT_50_ that was used for the clinical phase I study.

Plasma viremia was determined up to d14 after challenge, and clinical parameters such as body temperature, hematology, and inflammatory responses were assessed up to d28. On d28 after challenge, NHPs were euthanized, and the presence of WT CHIKV RNA was analyzed in relevant tissues.

The μPRNT_50_ titer measured before challenge on d0 in each individual macaque was used to determine the neutralizing antibody titer required to protect from WT CHIKV induced viremia and fever.

### Transfer of VLA1553-specific serum in NHPs suppressed plasma viremia.

Prior to WT CHIKV challenge, the μPRNT_50_ titers were determined in NHP serum after human serum transfer. The μPRNT_50_ titer ranges determined after transfer of d28 ULS, LS, MS, and HS, as well as HS d14, MHS d84, MS d180 and HS d180, are shown in Table 1. The μPRNT_50_ titers determined for each individual animal are shown in Supplemental Table 1 (supplemental material available online with this article; https://doi.org/10.1172/jci.insight.160173DS1). For the determination of the μPRNT_50_ titer, a heterologous Asian CHIKV genotype was used, demonstrating the cross-neutralizing potential of VLA1553-induced antibodies, which is in agreement with previously published data from Roques and colleagues (16).

**Figure 2:**
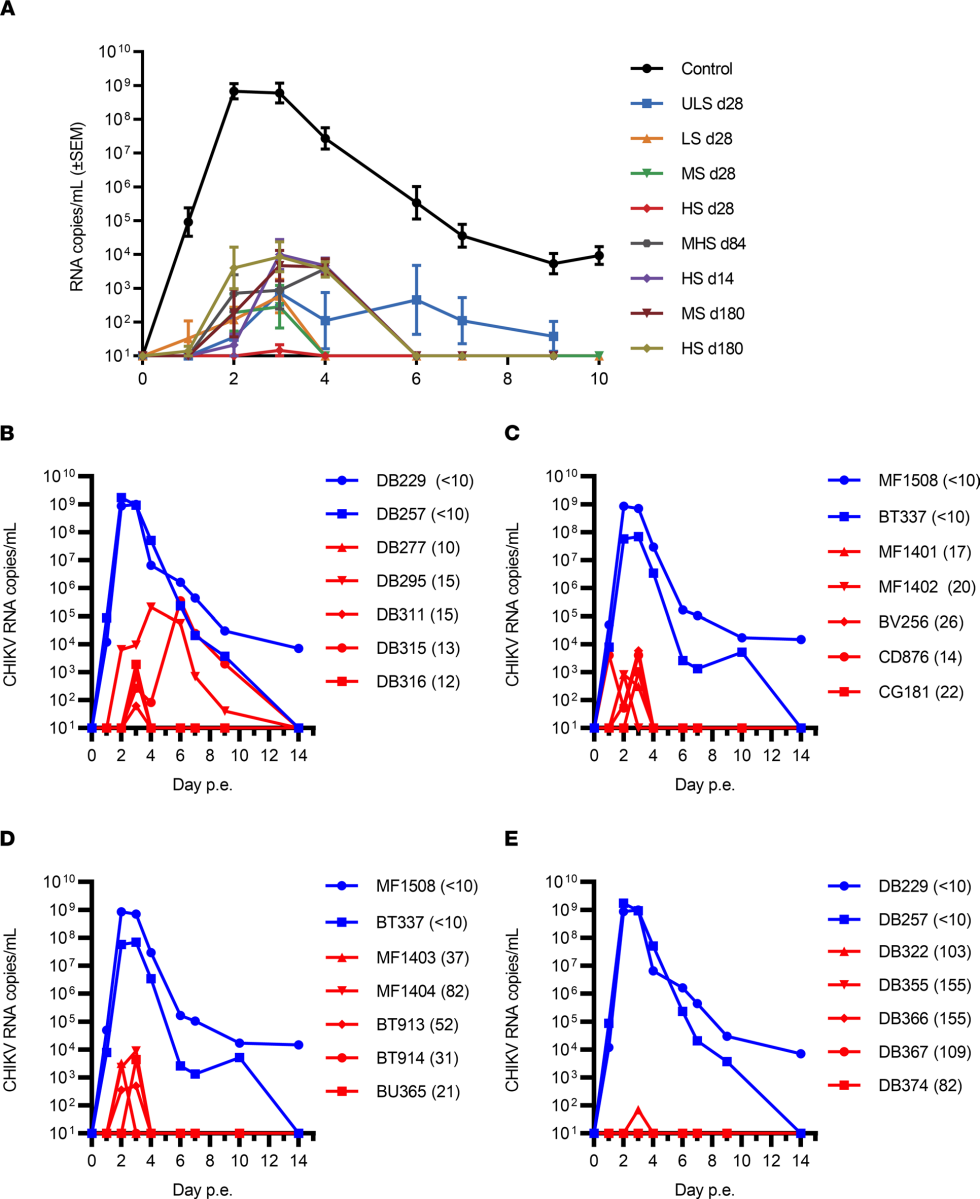
Viremia in plasma measured by qPCR. (**A**) Data represent mean viremia ± SEM per VLA1553 phase I serum treatment group and control animals. Viremia magnitude was significantly reduced (*P* < 0.001; repeated-measures 2-way ANOVA, mixed-effects analysis of log transformed data). (**B**–**E**) Viremia in individual animals showing viral load as determined after transfer of ULS d28 (titer range 10–15 μPRNT_50_) (**B**), LS d28 (titer range 14–26 μPRNT_50_) (**C**), MS d28 (titer range 21–82 μPRNT_50_ (**D**), and HS d28 (titer range 82–155 μPRNT_50_) (**E**). Control animals DB229 and DB257 are the same in **B** and **E**; control animals MF1508 and BT337 are the same in **C** and **D**. Control animals receiving nonimmune sera (<10 μPRNT_50_) are shown in blue; VLA1553-specific serum treated animals are shown in red. μPRNT_50_ titer prior to WT CHIKV exposure for the individual animal is shown in brackets. Plasma from d1 to d3 in **C** and **D** and from d1 and 2 in **B** and **E** were tested by classical qPCR (LLOQ, 5000 copies/mL [62.5 copies/rxn]; LLOD, 720 copies/mL [9 copies/rxn]). Samples from all other time points were tested by ultrasensitive qPCR (LLOQ, 500 CHIKV RNA copies/mL [42 copies/rxn]; LLOD, 60 CHIKV RNA copies/mL [5 copies/rxn]). p.e., postexposure.

To evaluate whether human VLA1553 phase I serum protected the animals from CHIKV infection, the plasma viral load was determined by quantitative PCR (qPCR) after WT CHIKV LR2006-OPY1 challenge. The challenge dose was close to 100 times AID_50_. Infection of cynomolgus macaques with LR2006-OPY1 at this virus dose typically results in plasma viremia that peaks at d2–d3 postexposure (p.e.) (16, 31–33). In control animals (*n* = 6) receiving human nonimmune serum prior to WT CHIKV challenge, CHIKV RNA was detected as early as d1 p.e. (Figure 2), peaked on d2–d3 p.e., and declined to undetectable levels by d14 p.e. (Figure 2 and Supplemental Figure 1). In all animals treated with VLA1553 phase I sera, the viremia peaks were overall strongly delayed. In addition, the viremia magnitude was significantly reduced by at least 3–5 logs. Similarly, the duration of viremia was also strongly reduced (Figure 2A).

Treatment with ULS d28 resulted in longer duration (3–4 days) and, in some animals, higher peaks (up to 3 × 10^5^ RNA copies/mL) of plasma viremia (Figure 2B). For animals treated with LS d28 or MS d28, viremia remained below 10,000 copies of CHIKV RNA/mL and lasted only 2–3 days or less in both treated groups (Figure 2, C and D). The mean of produced CHIKV RNA copies (as quantified by the AUC) was more than 5 logs lower than in the control group (LS d28, *P =* 0.0123; MS d28, *P =* 0.0099; Kruskal-Wallis test). Among all VLA1553 phase I serum-treated animals, only 1 animal treated with LS d28 showed a low level of viremia (4085 copies/mL) as early as on d1 (Figure 2C). One animal treated with MS d28 showed no viremia at all (Figure 2D). Transfer of d28 postvaccination sera with the highest titer (HS d28) resulted in a titer range in NHPs of 82–155 μPRNT_50_ and conferred sterilizing protection (no detectable CHIKV RNA) in 4 of 5 animals (Figure 2E). The fifth macaque showed a minimal level of viremia with 70 CHIKV RNA copies/mL, just above the lower limit of detection (LLOD) (60 CHIKV RNA copies/mL), at a single time point.

Viremia was observed in animals that received sera with intermediate μPRNT_50_ titers — HS d14, MHS d84, or MS d180 and HS d180 — but viremia was strongly reduced and lasted only 2–3 days (Supplemental Figure 1). In all animals, CHIKV RNA copy numbers at peak viremia were significantly decreased (*P =* 0.0078) by more than 4 logs compared with control animals. The μPRNT_50_ titers and peak viremia values for each individual animal as measured by qPCR (RNA copies per mL) are listed in Supplemental Table 1.

Importantly, no replicating WT CHIKV was detectable by 50% tissue culture infectious dose (TCID_50_) assay in any of the plasma samples from NHPs treated with VLA1553 phase I sera, no matter whether ULS, LS, MS, or HS was transferred, in contrast to NHPs receiving nonimmune sera that showed detectable infectious CHIKV (Supplemental Table 2).

### Determination of a threshold titer as a surrogate of protection against WT CHIKV viremia.

**Figure 3:**
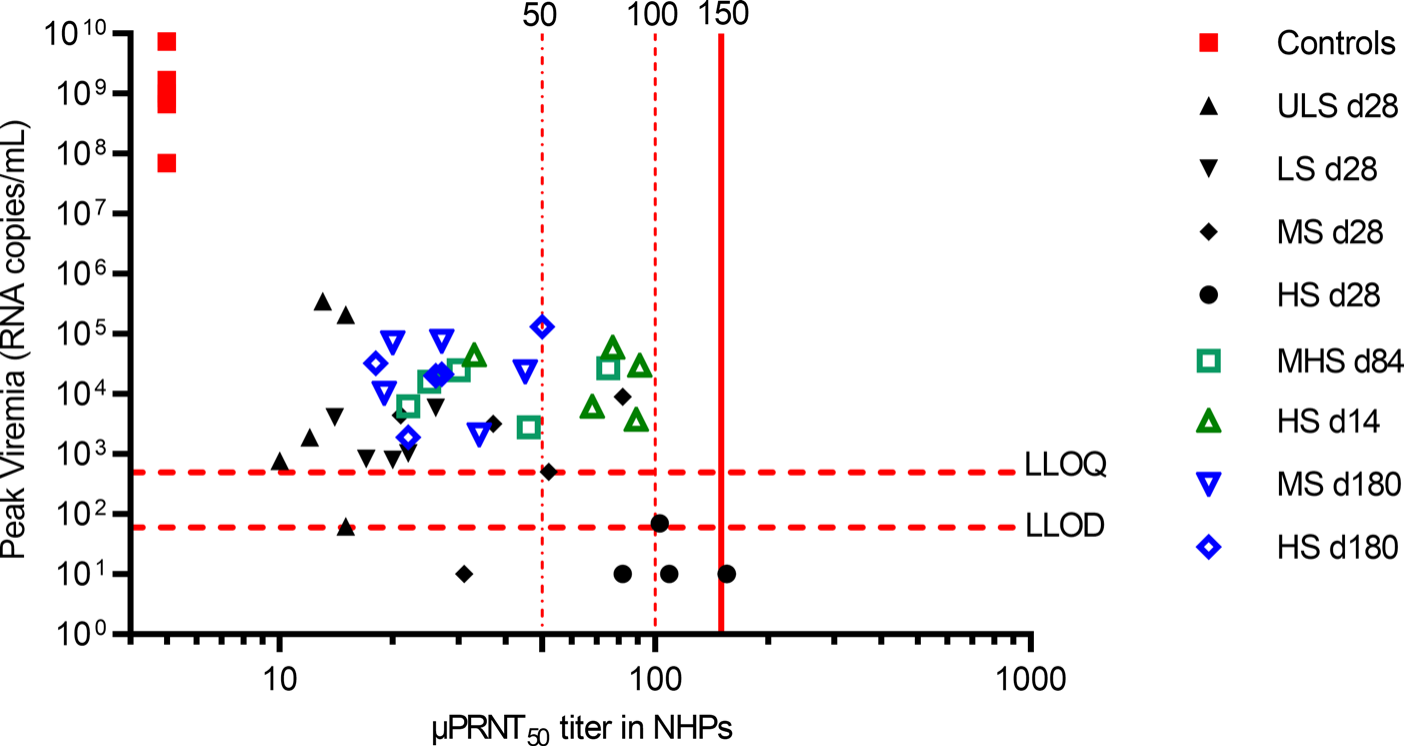
Peak viremia titers plotted against μPRNT_50_ titers. The μPRNT_50_ titers of the control NHPs receiving nonimmune serum were all negative (<10; Supplemental Table 1) and were, therefore, imputed to 5. Undetectable viremia titers were imputed to 10. Filled symbols denote VLA1553 phase I sera at d28; unfilled symbols denote sera obtained at d14, d84, and d180 after vaccination. For HS d28, 2 data points have identical μPRNT_50_ titers with undetectable RNA; they are, thus, represented by 1 data point. Horizontal red dotted lines show LLOQ (500 copies/mL) and LLOD (60 copies/mL) of the qPCR. Vertical red dotted lines show μPRNT_50_ titers of 50 and 100, while the vertical solid line shows the μPRNT_50_ titer of 150. ULS, ultra-low titer serum; LS, low titer serum; MHS, medium high titer serum; MS, medium titer serum; and HS, high titer serum.

In order to determine a surrogate of protection using the passive transfer of human sera in a NHP CHIKV challenge model, we considered as evidence of protection the lack of CHIKV RNA detection in the blood. CHIKV RNA was detected by a sensitive qPCR assay with a LLOD of 60 CHIKV RNA copies/mL (5 copies/reaction) and a lower limit of quantification (LLOQ) of 500 CHIKV RNA copies/mL (42 copies/reaction). Based on the data obtained in the NHP CHIKV challenge model (Figure 3 and Table 2), the threshold titer for protection was set at a μPRNT_50_ titer of ≥ 150 (Figure 3, solid red vertical line) at which none of the animals had CHIKV RNA that was detectable by qPCR.

Evaluating all NHPs of the study, 13 animals had a μPRNT_50_ titer ≥ 50 after VLA1553 phase I serum transfer and prior to WT CHIKV challenge (d0). At this titer, 4 animals showed no CHIKV RNA in serum (Figure 3, dotted vertical red line). Four animals in the study reached a μPRNT_50_ titer ≥ 100 after serum transfer, and 3 of these NHPs had no detectable CHIKV RNA after WT CHIKV challenge. When assessing animals that had a μPRNT_50_ titer of ≥ 150, none of the 2 NHPs showed any CHIKV RNA as detected by qPCR after WT CHIKV challenge (Figure 3, red solid vertical line). Thus, a μPRNT_50_ titer of ≥ 150 was proposed as a surrogate of protection.

### Passive transfer of VLA1553 phase I sera protected animals also against clinical symptoms.

In addition to the assessment of viremia, NHPs were also evaluated for clinical symptoms after WT CHIKV challenge. Besides the monitoring of body temperature, a full hematology analysis was performed.

### Protection against fever.

**Figure 4:**
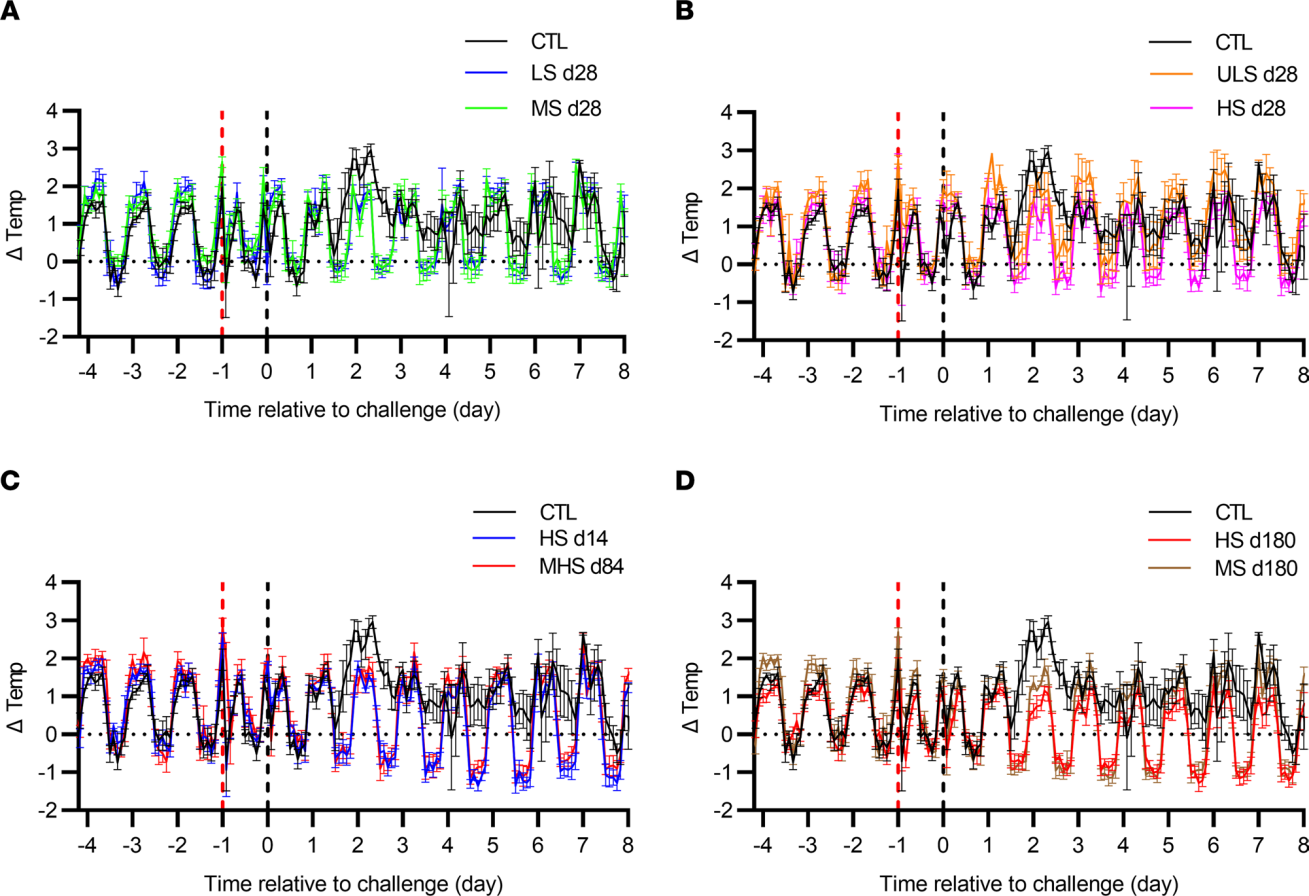
Body temperature after WT CHIKV challenge in control and VLA1553 phase I serum–treated NHPs. (**A**–**D**) Data represent mean ± SD of body temperature measured with implanted STAR ODDI chips every 2 hours for animals treated with LS d28 (in blue) and MS d28 (in green) (**A**), ULS d28 (in orange) and HS d28 (in pink) (**B**), HS d14 (in blue) and MHS d84 (in red) (**C**), and HS d180 (in red) and MS d180 (in brown) (**D**). Black lines show the control animals receiving human nonimmune serum. For all 4 panels, data for the same control animals are shown in comparison with the respective treated animals. Temperature was normalized to the night temperature (baseline on 10 consecutive nights; 10 p.m. to 6 a.m.) for each animal treated with d28 sera and to 3 consecutive nights for animals treated with d14, d84, and d180 serum as recorded chip temperature can also vary depending on animal size and exact location of the chip in the back. Temperature record from time of sampling was removed for clarity as temperature drops (around 10 a.m.) due to anesthesia were registered, except for the ones signing the serum injection (d–1) and virus exposure (d0, time 0). Remaining temperature drops in the middle of the daytime were due to nap periods. Mean ± SD were obtained from 5 animals except for 4 animals receiving LS d28 and for 3 animals receiving ULS d28 due to defective chips.

For the assessment of the body temperature after WT CHIKV challenge, s.c. implanted STAR ODDI chips recorded the body temperature of NHPs every 2 hours. For NHPs receiving nonimmune human sera prior to WT CHIKV exposure, fever was persisting from d1 to d7 (Figure 4, black lines). In contrast, animals receiving d28 VLA1553 phase I sera with either high, medium, or low titer did not show any fever at all (Figure 4, A and B; pink, blue, and green lines), despite the low level of viremia detected in some of the animals. However, in animals that have received the ULS d28, showing viral replication during 4–5 days after challenge, a slight increase of temperature was observed during nights 2 and 3 p.e., although this was not significant (Figure 4B, orange line). For all animals treated with HS d14 and MHS d84 (Figure 4C; blue and red panel, respectively), as well as with MS d180 and HS d180 (Figure 4D), despite showing some level of viremia, no fever was detected during the entire observation period.

### Protection against lymphopenia and neutrophilia.

Animals receiving the control nonimmune sera prior to exposure to WT CHIKV showed lymphopenia and neutrophilia. These clinical symptoms were consistent with the observed plasma viremia and increased body temperature, and they were synchronized with the CHIKV RNA peak (Supplemental Figure 2). In contrast, in all VLA1553 phase I serum–treated NHPs, such an effect was not observed, except for 1 animal treated with ULS d28 prior to WT CHIKV challenge that showed signs of low lymphopenia. This animal also showed the highest viremia peak at 3.5 × 10^5^ copies/mL among all animals treated with VLA1553 phase I serum (Supplemental Table 1). In addition, no significant modifications of other blood parameters were observed in any of the VLA1553 phase I serum–treated animals. The cell blood count data were also in accordance with the CHIKV RNA levels that were at least 3–5 logs decreased, as compared with the control group (Figure 2A).

In conclusion, treatment of NHPs with human immune serum derived from the phase I study prior to WT CHIKV challenge protected almost all animals from development of any clinical signs of CHIK disease, while NHPs from the control groups treated with human nonimmune serum developed fever, lymphopenia, and neutrophilia upon WT CHIKV challenge.

### Protection against expression of inflammation markers.

In CHIKV infections of both humans and macaques, viral load and severity of disease are strongly correlated with the plasma increase of several cytokines such as IFNs, IL-6, IL-1RA, TNF-α, and MCP1 (31, 34–42). Therefore, the increase of inflammatory markers was analyzed in NHPs receiving either VLA1553 phase I or nonimmune serum prior to WT CHIKV challenge.

In animals from the control groups receiving human nonimmune serum, WT CHIKV challenge clearly induced signs of inflammation. Coinciding with the time of peak viremia, animals showed high concentration of Granzyme B, IL-1RA, MCP-1, IFN-α, and IL-8 (Supplemental Figure 3). A slight increase of TNF-α at d9 after challenge was also observed. In contrast, all VLA1553 phase I serum–treated NHPs were protected from strong inflammatory responses, with the exception of some animals receiving ULS d28 and LS d28, which expressed at least 1 inflammatory marker, albeit with a delay and at a lower level compared with animals in the control group. The cytokine and chemokine profiles are shown in Supplemental Figure 3.

### Protection against CHIKV RNA presence/persistence in tissues.

In addition to analysis of plasma viremia, clinical symptoms, and inflammatory responses, the viral load in selected tissues was determined after euthanasia at d28 after WT CHIKV exposure. Total CHIKV RNA was determined using qPCR within a set of tissues: lymphoid tissues, liver, muscles (flexor digitorum profundus, extensor digitorum brevis), joints, brain, and reproductive tissue. In NHPs receiving nonimmune serum, CHIKV RNA was consistently detected in lymphoid tissues (lymph nodes and spleen) 28 days after challenge, sporadically in joints and reproductive tract, and in 1 animal in the liver. Furthermore, CHIKV RNA was consistently detected in the inoculated muscle (Supplemental Figure 4).

In contrast, animals treated with d28 VLA1553 phase I serum were fully protected from CHIKV persistence, which is in agreement with the low or undetectable viremia in these treatment groups. Only 3 of the 20 NHPs treated with d28 VLA1553 phase I serum showed detectable CHIKV RNA at 2–5 logs lower level than in control animals (comparison of peak viremia, *P* = 0.0009). In 1 animal receiving HS d28 (the same and only animal in the HS d28 serum treatment group with detectable but extremely low plasma viremia at 70 RNA copies/mL), CHIKV RNA was detectable in the axillary lymph node (<LLOQ). The other 2 animals received ULS d28, and in 1 animal, CHIKV RNA was detectable in axillary and inguinal lymph nodes; in the other one, it was detected in the spleen (Supplemental Figure 4). The animal treated with ULS d28 and in which CHIKV RNA was detectable in axillary and inguinal lymph nodes, as well as the spleen, was the only NHP showing low signs of lymphopenia. It was also the animal with the highest viremia peak (3.5 × 10^5^ copies/mL) in the study among all animals treated with VLA1553 phase I serum (Figure 2 and Supplemental Figure 1). Among the NHPs treated with d14, d84, and d180 after vaccination VLA1553 phase I serum, only 1 animal receiving HS d14 was not protected from CHIKV RNA presence at d28, despite a comparable μPRNT titer, reduced viremia level, and lack of inflammatory responses after WT CHIKV challenge compared with animals from the same treatment group. NHPs receiving VLA1553 phase I serum from d14, d84, and d180 after vaccination (titer range 18–91 μPRNT_50_) were also protected from CHIKV RNA presence. Only in a few animals treated with serum from d14, d84, or d180 after vaccination, CHIKV RNA was sporadically detected in popliteal lymph nodes following WT CHIKV inoculation in the ankle (respective draining lymph node), as well as in reproductive tract and liver, but with RNA levels 1–4 logs lower than those in control animals.

### Evidence from seroepidemiology studies supports surrogate of protection.

The NHP passive transfer study allowed determining a surrogate of protection at a μPRNT_50_ titer of ≥ 150 based on protection from fever and the absence of CHIKV viremia after WT CHIKV challenge. To further support the defined surrogate, a panel of sera from a seroepidemiology study from the Philippines published by Yoon and colleagues (30, 43) was analyzed. Yoon and colleagues evaluated acute febrile illnesses via community-based active surveillance over a period of 12 months in 853 subjects with CHIKV infection and performed PRNT with blood samples obtained at enrolment and at 12 months after enrolment. The presence of detectable preexisting CHIKV neutralizing antibodies was associated with a decreased risk of PCR-confirmed symptomatic CHIKV infection. The authors concluded that a baseline CHIKV PRNT_80_ titer ≥ 10 was associated with 100% (95% CI, 46.1–100.0) protection from symptomatic CHIKV infection. In a follow-up study, it was shown that the presence of preexisting CHIKV neutralizing antibodies correlated with a decreased risk of both symptomatic CHIKV infection and subclinical seroconversion (43).

**Figure 5:**
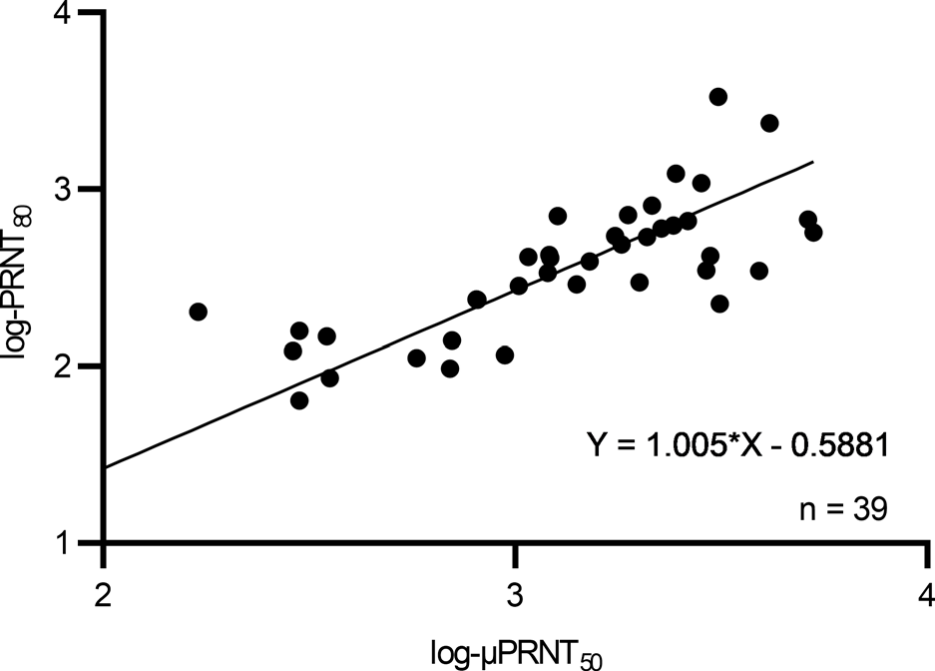
Linear regression of neutralization antibody titer using Deming regression analysis. Log transformed data of μPRNT_50_ versus PRNT_80_ shown.

The aim of the following analysis was to establish a correlation between neutralizing antibody titers measured by Yoon and colleagues (30) in a classical PRNT and the μPRNT, which was used to determine neutralizing activity of VLA1553-specific antibodies in the NHP passive protection model and in the ongoing VLA1553 Phase 3 clinical trials. Thus, the human serum samples as published by Yoon and colleagues and obtained from Armed Forces Research Institute of Medical Sciences (AFRIMS), Walter Reed Army Institute of Research (WRAIR) no. 1833 study (Prospective Cohort Study of Influenza and Dengue Infection in Children and Adults, Cebu City, Philippines), were analyzed in the μPRNT. In total, sera from 33 subjects (covering the observed neutralizing antibody titer range) with matching samples from year 1 and year 2 of the longitudinal seroepidemiological study were tested in the μPRNT using the CHIKV strain 181/clone 25, a live-attenuated derivative of Southeast Asian human isolate strain AF15561. The neutralization antibody titer showed a correlation between both test methods (Figure 5 and Supplemental Table 3). All sera that tested negative in AFRIMS PRNT_80_ (*n* = 27) were confirmed negative in the μPRNT and vice versa. In an analysis of all positive sera (*n* = 39), the geometric mean ratio (GMR) of μPRNT_50_/PRNT_80_ was at 3.73 (99% CI, 2.86–4.87). Applying the 99% upper CI, 4.87, the protective PRNT_80_ titer of ≥ 10 determined by Yoon and colleagues (30, 43) would translate into a μPRNT_50_ titer of 48.7. Even applying most stringently the highest μPRNT_50_/PRNT_80_ ratio for an individual sample of 13.93, the protective PRNT_80_ titer of ≥ 10 would translate into a μPRNT_50_ titer of ≥ 139.3 (Table 3). These results show that the proposed correlate of a PRNT_80_ ≥ 10 determined in the seroepidemiological study from Yoon and colleagues (30, 43) is in agreement with the surrogate of protection, a μPRNT_50_ titer ≥ 150, established in the NHP passive transfer study using VLA1553 phase I serum samples.

## Discussion

We used a WT CHIKV challenge model in NHPs in order to assess the effectiveness of human VLA1553 immune sera derived from phase I to protect animals from CHIK disease. The WT CHIKV challenge dose used for this study was within the range of an infectious CHIKV dose likely delivered by mosquitoes (44), thus mimicking natural infection. Animals treated with human nonimmune serum developed symptoms of CHIK disease, such as high plasma viremia, presence of replication-competent CHIKV in plasma, and clinical symptoms — e.g. fever, neutrophilia, and lymphopenia; increased expression of inflammatory cytokines typically associated with WT CHIKV infection, as well as presence of CHIKV in tissues, such as lymph nodes, spleen, joints, liver, and reproductive tract). The observations in control animals were in agreement with symptoms of WT CHIKV infection in NHPs as previously published (31).

In this study, we demonstrated that NHPs were protected from WT CHIKV-induced viremia, fever, and further clinical signs of CHIK disease upon transfer of VLA1553 phase I serum obtained from subjects vaccinated in the phase I clinical trial (17). In addition, we determined a threshold neutralizing antibody titer (μPRNT_50_ ≥ 150) as a surrogate of protection, at which titer none of the animals showed any detection of CHIKV RNA in the plasma or any fever after challenge with WT CHIKV.

When VLA1553 phase I serum of varying titers was transferred to NHPs prior to WT CHIKV challenge, animals showed a highly significant decrease of viral RNA of at least 3–5 logs, and transfer of the highest titer human sera resulted in a complete lack of CHIKV RNA detection. Furthermore, the duration of viremia was strongly reduced, and replicating CHIKV in plasma could not be detected in any of the animals that received VLA1553 phase I serum. The transfer of VLA1553 phase I serum resulted in protection from clinical signs of CHIK disease, including fever or modification of blood parameters, and had a major effect on the inflammatory cytokine responses and presence of CHIKV in tissues. Thus, the transfer of human VLA1553 phase I sera at various titers allowed us to determine a threshold neutralizing antibody titer, required to provide protection against CHIK viremia and disease.

The surrogate of protection, a μPRNT_50_ titer ≥ 150 that is reasonably likely to predict clinical benefit in humans, was determined using a stringent approach. We only considered animals protected when no CHIKV RNA was detectable in plasma after challenge, and we applied a sensitive qPCR that was able to detect down to 60 copies of CHIKV RNA per mL of plasma (LLOD). Considering that the qPCR not only detects live, replicating virus, but that it also detects nonviable virus particles, it is important to note that — for RNA viruses — a single infectious viral particle as measured by TCID_50_ assay translates to numerous RNA copies as determined by qPCR. This is evidenced by several reports on the efficiency of virus replication in relation to the number of detectable RNA copies. Vanlandingham and colleagues have shown for the La Reunion OPY1 CHIKV strain that 200 genome equivalents/mL corresponded to 1 TCID_50_/mL (45). Carletti and colleagues have demonstrated a direct correlation of log-transformed viral titers (TCID_50_/mL) and RNA copies/mL with a conversion factor of approximately 500 (46).

In our studies, a conversion of 1 TCID_50_/mL to 200 copies/mL was determined for NHP plasma samples (ref. 16 and unpublished data). Considering this conversion factor of 200, the qPCR as applied in our study had a LLOD allowing the detection of less than the equivalent of a single infectious CHIKV particle per mL. In addition, at the highest viremia level of 3.5 × 10^5^ copies/mL detected in VLA1553 phase I serum–treated NHPs, no clinical signs of CHIK disease were observed and also no live infectious virus was detected in plasma samples by TCID_50_ assay. This showed that, at μPRNT_50_ titers between 10 and 150 prior to challenge, NHPs were fully protected against CHIKV infection–associated fever (and modification of blood parameters), despite showing low levels of viremia as determined by qPCR. Due to this approach for determination of the threshold neutralizing antibody titer, we consider the surrogate of protection — a μPRNT_50_ titer of ≥ 150 based on the lack of detectable CHIKV RNA — as conservative, as it is considerably higher than the titer required to protect animals from clinical signs of CHIK disease, such as fever and the detection of replicating CHIKV by TCID_50_ assay.

The surrogate of protection as determined by our passive transfer study was further supported by the data generated with sera derived from a seroepidemiology Philippine Cohort study (30, 43). The authors concluded that detectable CHIKV neutralizing antibody titer at baseline may correlate with protection from symptomatic CHIKV infection and subclinical seroconversion, supporting the potential use of the neutralizing antibody titer as a surrogate endpoint for protection. The analyses of the sera in our μPRNT showed that the proposed correlate of protection, a PRNT_80_ titer ≥ 10, can be translated even when applying the most stringent conditions into a μPRNT_50_ titer ≥ 139.3. This confirms that the surrogate of protection established in the NHP study was conservatively set at a μPRNT_50_ titer ≥ 150.

We determined the surrogate of protection based on data from 2 independent sources of evidence, the NHP passive transfer study, and data using sera from a seroepidemiology study; this alleviates concerns about relying on the results of a single animal experiment or only on seroepidemiological data. While data derived from humans in prospective seroepidemiological studies may have certain limitations, such as surveillance and testing methods to identify clinical cases of CHIK disease or the detection of baseline CHIKV-neutralizing antibody titers caused by recent CHIK infection, the NHP passive transfer model also has limitations. Nevertheless, the applied cynomolgus macaque (*M. fascicularis*) NHP model is considered as the most relevant model for CHIK disease, since the immune and pathological responses of NHPs to CHIKV are similar to those seen in human infections (31, 47).

Our approach to define a serological surrogate of protection based on data using human sera derived from seroepidemiological studies and passive transfer studies in NHPs is in agreement with the consensus of the 158th Meeting of the Vaccines and Related Biological Products Advisory Committee (VRBPAC) (48). Consequently, in preparation for the Phase 3 study, our proposed surrogate of protection of a μPRNT_50_ titer ≥ 150 was accepted by the FDA as a measure of the seroprotective neutralizing antibody titers, likely to predict clinical benefit, as induced by the VLA153 vaccine.

In conclusion, we have provided evidence that human sera from VLA1553 vaccine recipients can protect NHPs against infection and CHIK disease. Furthermore, we have determined a surrogate of protection based on 2 approaches from independent sources of evidence, a passive transfer study in NHPs using human VLA1553 phase I sera and data generated with human sera from a seroepidemiology study. The surrogate threshold titer indicative of protection in the NHP model has been established using stringent criteria. While protection from clinical CHIK symptoms was seen already at lower VLA1553 phase I titer levels, the surrogate titer of μPRNT_50_ ≥ 150 was determined based on the requirement for sterilizing immunity in animals. Thus, our study provides a surrogate of protection reasonably likely to predict clinical benefit for the further clinical evaluation of the single shot live-attenuated CHIK vaccine.

## Methods

### Animals and ethics.

Forty-six CHIKV-naive cynomolgus macaques (*M. fascicularis*), specifically dedicated to this study, were imported from international accredited breeding facilities in Mauritius. They were selected to provide homogenous groups in terms of age, weight, and MHC class I and class II haplotype when possible. Macaques were housed within the animal biosafety level 3 (ABSL3) facility at CEA, Fontenay-aux-Roses, and handled according to the directive 2010/63/CE and French law “décret 2013-118 from February 1st 2013.” Handling, social grouping, and enrichment were performed in accordance with institutional guidelines (CEA [French Alternative Energies and Atomic Energy Commission], Department IDMIT).

### General study design.

Animals were sedated with Tiletamine/Zolazepam (Zoletil, Virbac; 5 mg/kg [0.05 mL/kg, i.m. route]) before handling. Eight groups of NHPs received VLA1553 phase I serum pools administered in 4 rounds: 12 NHPs in round 1 and 12 NHPs 2, with 2 groups of 5 animals receiving VLA1553 phase I serum from d28 after vaccination and 2 animals receiving nonimmune serum. Eleven NHPs were included in round 3 as well as round 4, with 2 groups of 5 animals receiving VLA1553 phase I serum from d14, d84, and d180 after vaccination and 1 animal receiving human nonimmune serum in each round. In the first round, serum transfer was performed with LS d28 and MS d28, and after the outcome of the first round was known, serum transfer with ULS d28 and HS d28 was performed. In a confirmatory third round, passive transfer of HS d14 and MHS d84 was performed. In the fourth round, NHPs received MS d180 and HS d180 to assess persistence of the antibody response after VLA1553 vaccination. NHPs (*M. fascicularis*) received 3 mL of human serum per kg of weight, injected i.v. The control groups receiving human nonimmune serum consisted of 3 males and 3 females, whereas 20 males and 20 females were included in the experimental groups receiving VLA1553 phase I serum. Animals receiving serum pools from d28 after VLA1553 vaccination were all females except for 1 animal. Animals receiving serum pools from d14, d84, and d180 after VLA1553 vaccination were all males. Just prior to challenge, a blood sample was drawn from each animal to measure anti-CHIKV neutralizing antibody titers using a μPRNT that was also applied for clinical testing. NHPs were challenged with WT CHIKV LR2006-OPY1 s.c. in the left wrist (round 1 and 2 animals) or left ankle (round 3 and 4 animals) with 100 AID_50_ corresponding to 7000–10,000 PFU (33). After challenge, safety parameters (clinical assessment), plasma viremia, signs of CHIKV infection, and relevant hematological parameters were analyzed. d28 after challenge, NHPs were humanely euthanized, and blood as well as various organs/tissues were collected from all animals — e.g., in order to determine presence of CHIKV RNA.

### Study procedures.

Clinical examinations (including swelling of wrist and ankle [left/right] or signs of other inflammatory diseases) were performed until d28. Rectal temperature and weight were recorded 10 minutes after sedation, before immunization, challenge, or bleeding.

In addition, body temperatures were recorded using STAR ODDI chips (www.star-oddi.com) that were implanted s.c. in the upper back, between scapulas 3 weeks before human serum transfer and WT CHIKV exposure. These devices recorded body temperature every 2 hours.

They were removed at euthanasia to collect data. At d28 after WT CHIKV exposure, sedated animals were euthanized by i.v. injection of a lethal dose of pentobarbital.

### Selection, preparation, and pooling of human sera used for passive transfer in NHPs.

Selection of human VLA1553 sera from phase I was done based on neutralizing antibody titers determined in a microneutralization (μNT) assay. Briefly, the μNT detects neutralizing antibodies against infection of Vero cells with the CHIKV Δ5nsP3 vaccine virus strain (VLA1553), and the neutralizing antibody titer was determined as the serum dilution, which caused 50% protection from cell death (17). For control groups, serum from d0 (before VLA1553 vaccination) of the 120 subjects without detectable anti-CHIKV antibodies enrolled in the study were pooled. All serum pools were heat inactivated prior to use. The titers of VLA1553 phase I serum pools derived from vaccinated subjects before transfer in NHPs are shown in Table 1.

### Quantification of VLA1553-specific neutralizing antibody titers in NHP serum.

CHIKV neutralizing antibody titers of serum samples obtained from NHPs after human serum transfer and prior to WT CHIKV challenge were analyzed by μPRNT using a serially passaged, live-attenuated CHIKV vaccine (TSI-GSD-218 or 181/clone 25) developed by the WRAIR. The cut-off of the assay was defined as μPRNT_50_ of 10 during assay qualification. This assay was performed by NEXELIS (former NeoMed Laboratory Holdings Inc.) and was also used as clinical assay for Valneva′s CHIK vaccine candidate VLA1553.

In brief, seven 2-fold serial dilutions of heat-inactivated serum samples were prepared in round-bottom 96-well plates. CHIKV 181/25 at 100 PFU/well was added sequentially to the serum dilutions and incubated at 37°C and 5% CO_2_ for 60 minutes. Serum-virus complexes were then transferred onto plates, previously seeded overnight with Vero cells and were incubated at 37°C and 5% CO_2_ for 60 minutes. Then, the serum-virus complexes were removed and a virus-maintenance medium containing methyl cellulose was added to the wells. After an overnight incubation at 37°C and 5% CO_2_, cells were fixed. Following cell permeabilization, indirect immunostaining was performed. For detection, a primary mouse anti–CHIKV E2 protein antibody and a secondary anti-mouse antibody were used. Substrate was added to the plates and incubated for 10 minutes in the dark. Images from each well were acquired by an automated microscope system (ScanLab reader), and the number of plaques was counted using AxioVision. The number of resulting plaques in the wells is inversely proportional to the level of functional antibodies present in the serum, which is directly proportional to the immunological response of the subject.

The cut-off of the assay was defined as μPRNT_50_ of 10 during assay qualification, and the LLOQ was confirmed at 20 during method validation.

### Biochemical and immunovirological follow-up.

Animal blood was sampled twice at base line and then each day from d1 after serum infusion until challenge. Blood was, in addition, sampled 10 minutes before challenge and on d0 after challenge. Then, it was sampled daily up to d4;on d6, d7, d9, or d10; and d14 and d28 after challenge.

Full hematology (complete blood count) was performed using a HMX A/L (Beckman Coulter) on blood collected in EDTA tubes. The same blood samples were used for plasma viral load determination and cytokine quantification.

### CHIKV quantification in NHP plasma.

Plasma CHIKV viral loads were determined daily from d1 until day 4; on d6, d7, and d9 or day d10 after challenge for all groups; and on d14 after challenge for animals receiving d28 serum using qPCR essentially as already described (16, 31). Briefly, CHIKV RNA was extracted either from 100 μL of NHP plasma with Nucleospin 96 Virus Core Macherey-Nagel kit (no. 740452.4) for classical qPCR or from 500 μL NHP plasma using the QIAamp UltraSens Virus Kit (Qiagen, 53704) for ultrasensitive (US) qPCR for high and low titer samples, respectively. qPCR was performed on 10 μL of purified RNA (from 80 μL eluted volume in classical and 60 μL eluted volume in US qPCR) using the Superscript One-Step qPCR kit (Invitrogen, 11732088) according to manufacturer recommendations, with CHIKV-specific primers ChikF1 (forward; AAG CTC CGC GTC CTT TAC CAA G) and ChikR1 (reverse; CCA AAT TGT CCT GGT CTT CCT) and the CHIKV-specific probe (FAM-CCA ATG TCT TCA GCC TGG ACA CCT TT-BHQ1). qPCR was performed using a CFX96 Touch Real-Time PCR Detection System (Bio-Rad). All amplifications were performed in duplicate for classical and in triplicate for US qPCR methods.

For CHIKV quantification in NHP plasma using classical qPCR, the LLOQ was determined at 5000 copies/mL (62.5 copies/rxn). The sensitivity of the assay (LOD_95_) has been demonstrated to be 720 copies/mL (9 copies/rxn) and is, thus, also the LLOD. For US qPCR, the LLOQ was determined at 500 copies/mL (42 copies/rxn) and the sensitivity (LOD_95_) was demonstrated to be 60 copies/mL (5 copies/rxn), thus also constituting the LLOD.

### Cytokine and chemokine profiles of inflammation and innate immune responses.

The cytokine and chemokine profiles in plasma of NHPs were analyzed using a bead-based multiplex assay. Measurements were performed daily from d1 until d4; on d6, d7, and d9 or d10; and on d14 and d28 after challenge. 

With the Milliplex system (MILLIPLEX MAP Non-Human Primate Cytokine Magnetic Bead Panel, MilliporeSigma), IFN-γ, IL-2, IL-4, IL-6, IL-8, IL-10, IL-12/IL-13 (p40), IL-18, and TNF-α levels were measured. Using the ProcartaPlex system (eBioscience, Thermo Fisher Scientific) Granzyme B, IFN-α, IL-1β, IL-1RA, IL-12 (p70), IL-15, IL-17A, MCP-1, and MIP-1β levels were determined.

The assays were performed according to the manufacturer’s instructions. The various time points were evaluated 1 time. Seven standards as well as negative controls provided in the kits were included. In addition, an in-house internal control was generated by pooling supernatants of *M. fascicularis* PBMCs that were cultivated in RPMI + 10% FCS and activated with either PMA-ionomycin, concanavalin A, or LPS, in order to assess the efficiency and reproducibility of the kit.

### Tissue collection and quantification of WT CHIKV in tissues.

On d28 after WT CHIKV exposure, all animals were euthanized and tissues were collected. Tissue samples were snap frozen with dry ice for subsequent total RNA extraction. The following tissues were analyzed: lymphoid tissues (spleen, axillary, inguinal, and popliteal lymph nodes), liver, muscles (flexor digitorum profundus for animals treated with serum from d28 after VLA1553 vaccination, as the WT CHIKV inoculation site was the left wrist; extensor digitorum brevis for animals treated with serum from d14, d84, and d180 after vaccination, as the WT CHIKV inoculation site was the left ankle), joints (left and right knee capsule), brain (cerebellum, mesencephalon, and frontal encephalon), and reproductive tissue.

### Tissue lysis and total RNA extraction.

Tissue lysates were generated by mechanical disruption of tissue samples in NucleoZOL buffer (Macherey-Nagel, 740404.200). All lysed tissues were further processed with a Precellys system, using tubes with ceramic beads for soft tissues (Bertin Technologies, 03961-1-0092) and tubes with metal beads for hard tissues (Bertin Technologies, 03961-1-0012). The tissue lysates were diluted to 100 mg/mL in NucleoZOL buffer for further use for RNA extraction and qPCR.

### CHIKV RNA quantification in tissue.

CHIKV quantification in tissues was essentially performed as described by Labadie et al. (31) where CHIKV RNA qPCR quantification cycle (Cq) measurements were compared with cellular GAPDH RNA (31). Total RNA was extracted using the Nucleospin 96 RNA Core kit (Macherey-Nagel, 740466.4) following the manufacturer instructions. Per extracted tissue sample, relative qPCR was carried out in duplicate simultaneously using the CHIKV specific primers and FAM-Probe (as described for CHIKV quantification in plasma) and a Cy5 GAPDH–specific probe. For each qPCR, CHIKV, and GAPDH, a standard curve was included. All reactions were approximately 100% efficient; thus, each CHIKV sample was normalized using a GAPDH sample, and the relative copy number for each sample was calculated with the following equation: Relative copy number = 2 – (Cq CHIKV – Cq GAPDH).

CHIKV RNA quantification data were expressed as the ratio of CHIKV RNA to cellular GAPDH RNA. LLOD depended on the amount of GAPDH RNA within the sample and on the quantity of CHIKV RNA (35–38 Cq), which varied from one tissue to another, from 3 × 10^-7^ for muscle to 6 × 10^–5^ for joints.

To illustrate the level of detectable RNA in tissue, a calculation of the CHIKV genomic RNA/ng total RNA in each sample was performed. Total RNA was quantified per tissue sample using UV spectroscopy (absorbance at 260 and 280 nm). The calculation took into account the total RNA used in each amplification round. Per sample and assay, 500 ng of total RNA were used; thus, the GAPDH RNA amount/500 ng total RNA was determined, and the ratio of CHIKV RNA/ng total RNA was calculated accordingly. The samples from the standard curve were also normalized to 500 ng of total RNA (50 ng/μL, 10 μL per assay) from tissues per PCR; thus, the final CHIKV copies of the standard ranged from 1,730,000 to 207 copies.

### Statistics.

Krustal-Wallis test was used for multiple comparisons. Repeated-measures 2-way ANOVA followed by Bonferroni’s multiple-comparison test was used for time point analyses, and Deming regression was used for comparison of data from different neutralization assays. The statistical analyses were performed with GraphPad Prism 9.2 (GraphPad Software). Differences between groups were considered significant when *P* < 0.05.

### Study approval.

All procedures were validated and approved by the ethic committee from CEA no. 44 with decision no. A18-051 and validated by the French MENESR under the no. APAFIS 16755-2018091711094093v2 from November 26, 2018

## Supplementary Material

Supplemental data

## Figures and Tables

**Table 1:**
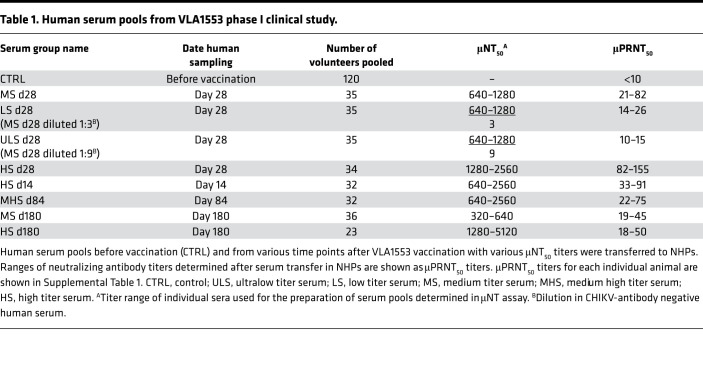
Human serum pools from VLA1553 phase I clinical study.

**Table 2:**
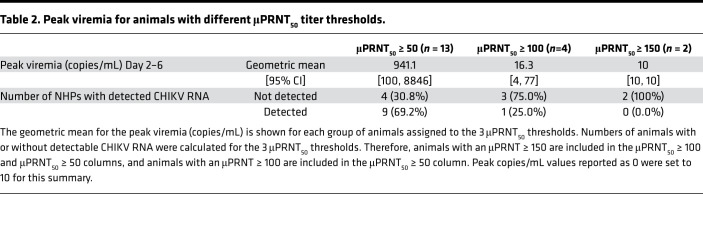
Peak viremia for animals with different μPRNT_50_ titer thresholds.

**Table 3:**
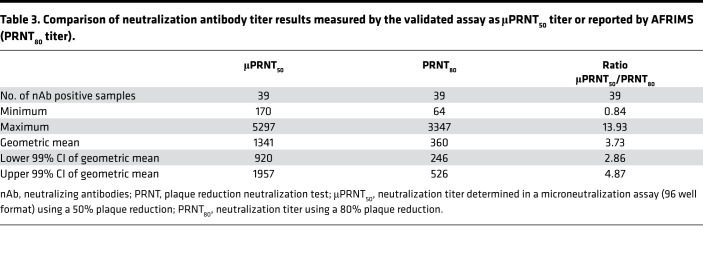
Comparison of neutralization antibody titer results measured by the validated assay as μPRNT_50_ titer or reported by AFRIMS (PRNT_80_ titer).

## Version 1 06/14/2022

In-Press Preview

## Version 2 07/22/2022

Electronic publication
