# Supplementary material for: Effectiveness of CHIKV vaccine VLA1553 demonstrated by passive transfer of human sera
Source: JCI Insight. 2022 Jul 22;7(14):e160173. doi: 10.1172/jci.insight.160173 (PMC9431671; doi:10.1172/jci.insight.160173)
Supplement: Supplemental data [file jciinsight-7-160173-s115.pdf]

Supplemental material

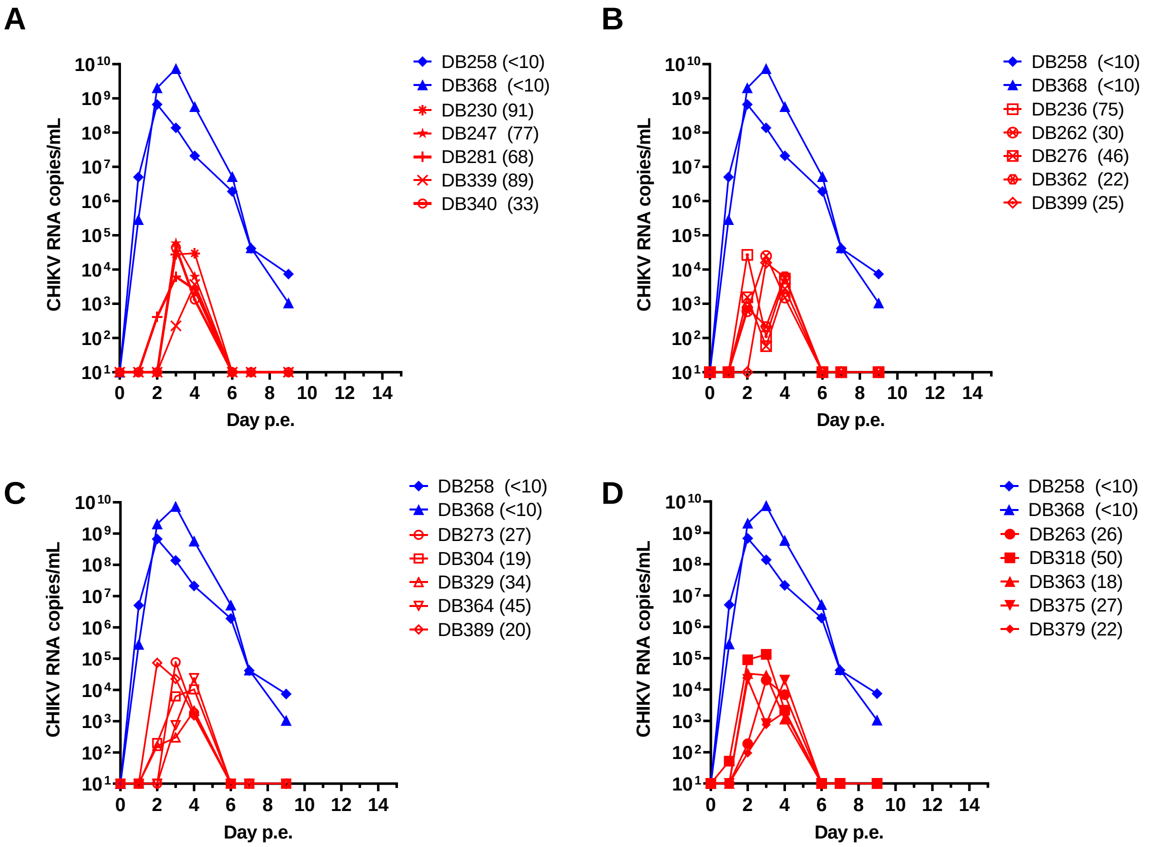

Figure S 1: Plasma viral load of individual animals. Viral load was determined in individual animals. The same control animals DB258 and DB368 receiving non-immune sera (<10 µPRNT$_{50}$) are shown for all four panels in blue. The other animals receiving VLA1553-specific serum **A.** HS d14 (titer range 33-91 µPRNT$_{50}$) **B.** MHS d84 (titer range 22-75 µPRNT$_{50}$) **C.** MS d180 (titer range 19-45 µPRNT$_{50}$) and **D.** HS d180 (titer range 18-50 µPRNT$_{50}$) are shown in red. µPRNT$_{50}$ titer for the individual animal is shown in brackets. LLOQ: 500 RNA copies/mL, LLOD: 60 RNA copies/mL (5 copies/reaction). p.e.: post-exposure.

Table S 1: Neutralizing antibody titers (µPRNT$_{50}$) of individual NHPs and peak viremia titers (copies/mL) after WT CHIKV challenge. µPRNT$_{50}$ titers prior to WT

CHIKV challenge were measured by Nexelis using an Asian genotype, the live-attenuated CHIKV vaccine strain TSI-GSD-218 or 181/clone 25 developed by the Walter Reed Army Institute of Research (WRAIR, US). Viremia was determined using RT-qPCR.

		**Day -1 (*Day -6)**	**Day 0**	**Day 2-6**	
**Serum transferred**	**Sample ID**	**μPRNT$_{50}$ titer**	**μPRNT$_{50}$ titer**	**Viremia peak (copies/mL)**	**Day of peak viremia**
Control	BT337	<10	<10	7.00×10^7	Day 3
	MF1508	<10	<10	8.5×10^8	Day 2
	DB229	<10	<10	1.00×10^9	Day 3
	DB257	<10	<10	1.70×10^9	Day 2
ULS d28	DB277	<10	10	7.79×10^2	Day 3
	DB295	<10	15	2.10×10^5	Day 4
	DB311	<10	15	6.20×10^1	Day 3
	DB315	<10	13	3.50×10^5	Day 6
	DB316	<10	12	1.90×10^3	Day 3
LS d28	MF1401	<10	17	8.35×10^2	Day 2
	MF1402	<10	20	8.07×10^2	Day 2
	BV256	<10	26	5.87×10^3	Day 3
	CD876	<10	14	4.08×10^3	Day 3
	CG181	<10	22	1.04×10^3	Day 3
MS d28	MF1403	<10	37	3.22×10^3	Day 2
	MF1404	<10	82	8.96×10^3	Day 3
	BT913	<10	52	5.07×10^2	Day 3
	BT914	<10	31	0	Day 3
	BU365	<10	21	4.45×10^3	Day 3
HS d28	DB322	<10	103	7.00×10^1	Day 3
	DB355	<10	155	0	Day 3

		Day -1 (*Day -6)	Day 0	Day 2-6	
**Serum transferred**	**Sample ID**	**μ$PRNT_{50}$ titer**	**μ$PRNT_{50}$ titer**	**Viremia peak (copies/mL)**	**Day of peak viremia**
	DB366	<10	155	0	Day 3
	DB367	<10	109	0	Day 3
	DB374	<10	82	0	Day 3
MHS d84	DB236	<10*	75	2.69×10^4	Day 2
	DB262	<10*	30	2.48×10^4	Day 3
	DB276	<10*	46	2.78×10^3	Day 4
	DB362	<10*	22	6.22×10^3	Day 4
	DB399	<10*	25	1.59×10^4	Day 3
HS d14	DB230	<10*	91	2.98×10^4	Day 4
	DB247	<10*	77	5.87×10^4	Day 3
	DB281	<10*	68	6.19×10^3	Day 3
	DB339	<10*	89	3.69×10^3	Day 4
	DB340	<10*	33	4.39×10^4	Day 3
Control	DB258	<10*	<10	6.70×10^8	Day 2
	DB368	<10*	<10	7.40×10^9	Day 3
MS d180	DB273	<10*	27	7.70×10^4	Day 3
	DB304	<10*	19	1.04×10^4	Day 4
	DB329	<10*	34	2.14×10^3	Day 4
	DB364	29	45	2.38×10^4	Day 4
	DB389	<10*	20	7.31×10^4	Day 2
HS d180	DB263	<10*	26	2.00×10^4	Day 3
	DB318	<10*	50	1.32×10^5	Day 3
	DB363	<10*	18	3.27×10^4	Day 2
	DB375	<10*	27	2.13×10^4	Day 2
	DB379	<10*	22	1.89×10^3	Day 4

Table S 2: $TCID_{50}$ titers at time of peak viremia and post peak viremia. NHP plasma samples from control animals receiving non-immune serum and VLA1553 Phase 1 serum treated animals were analyzed at peak viremia (day 2 and 3 p.e.), day 4 p.e. and post-peak viremia (day 6 and 7 p.e.). Replicating CHIKV was determined in a conventional Vero-cell based $TCID_{50}$ assays or with a pre-amplification step on mosquito cells for detection of replicating CHIKV in expected low titer samples.

Treatment group	Animals	Serum	Day p.e.	copies/mL	$TCID_{50}$/mL
Controls	BT337	human non-immune serum	2	5.79E+07	3.50×10^5 *
	BT337		2	5.80E+07	3.16×10^5
	BT337		3	7.00E+07	4.22×10^4
	BT337		4	3.50E+06	n.d.
	DB229		2	8.70E+08	2.37×10^6
	DB229		3	1.00E+09	7.50×10^5
	DB229		4	7.00E+06	6.25×10^3
	DB229		7	4.40E+05	n.d.
	DB258		6	1.92E+06	n.d. *
	MF1508		2	8.50E+08	1.78×10^6
	MF1508		6	1.70E+05	n.d.
	DB257		2	1.70E+09	2.37×10^6
	DB257		3	9.30E+08	1.78×10^6
	DB257		7	2.10E+04	n.d.
VLA1553-specific human serum	BV256	MS d28	3	5.87E+03	n.d. *
	MF1404	LS d28	3	8.96E+03	n.d. *
	DB295	ULS d28	3	9.30E+03	n.d. *
	DB295		4	2.08E+05	n.d. *

	DB295		6	5.52E+04	n.d. *
	DB315		7	2.46E+04	n.d. *
	DB315		9	1.98E+03	n.d. *
	DB230	HS d14	3	2.98E+04	n.d. *
	DB340		3	4.40E+04	n.d. *
	DB247		3	5.87E+04	n.d. *
	DB281		3	6.19E+03	n.d. *
	DB262	MHS d84	3	2.48E+04	n.d. *
	DB399		3	1.59E+04	n.d. *
	DB364	MS d180	4	2.38E+04	n.d. *
	DB304		4	1.04E+04	n.d. *
	DB363	HS d180	2	3.27E+04	n.d. *
	DB375		4	2.00E+04	n.d. *
	DB263		3	2.00E+04	n.d. *
	DB318		3	1.32E+05	n.d. *

p.e.: post-exposure; n.d.: not-detectable; HS: high titer serum; MHS: medium high titer serum; MS: medium titer serum; LS: low titer serum; ULS: ultra-low titer serum.

*, $TCID_{50}$ assay with a pre-amplification step on mosquito cells.

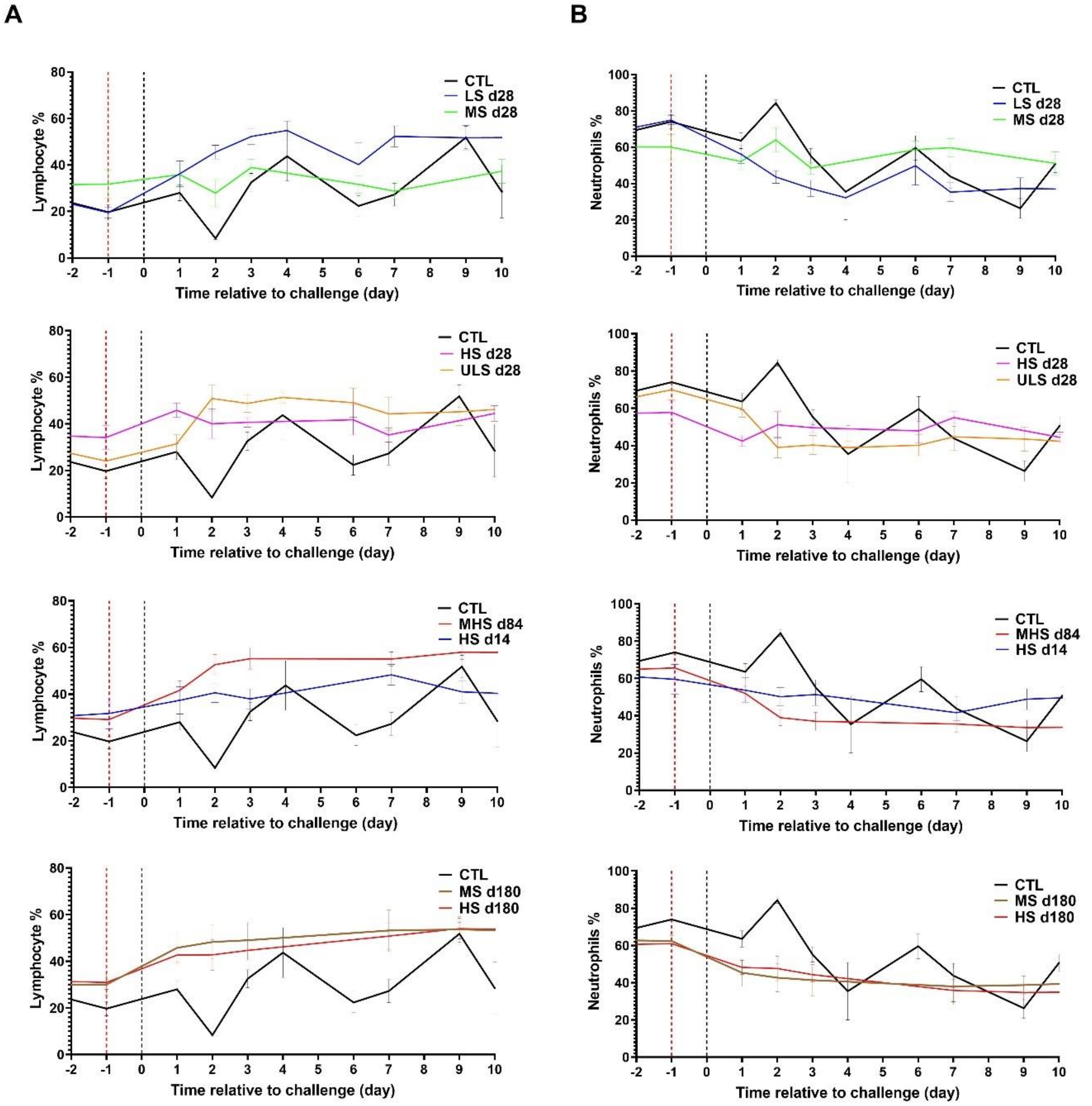
A
B
Lymphocyte %
Neutrophils %
Time relative to challenge (day)
CTL
LS d28
MS d28
HS d28
ULS d28
MHS d84
HS d14
MS d180
HS d180

Figure S 2: Cell blood counts post WT CHIKV challenge. Cell blood counts were compared between animals receiving VLA1553 Phase 1 serum and NHPs receiving human non-immune serum (CTL, black lines) prior to WT CHIKV challenge. Data represent Mean ± standard deviation of percentages of **A.** lymphocytes, the same six control animals (CTL) are shown on all four graphs and **B.** neutrophils within animals from each group, the same six control animals (CTL) are shown on all four graphs.

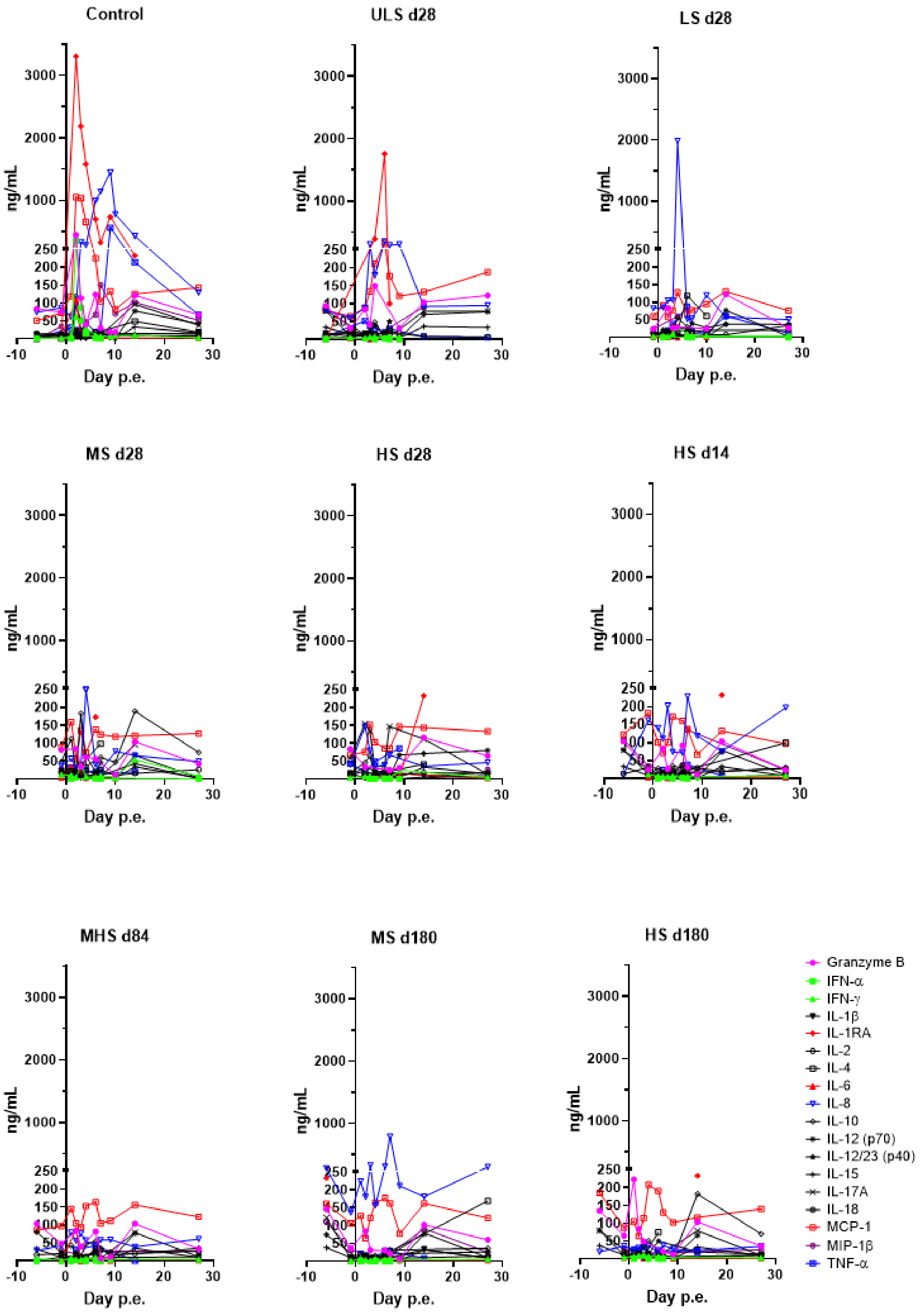

Control
ULS d28
LS d28
MS d28
HS d28
HS d14
MHS d84
MS d180
HS d180
ng/mL
Day p.e.
Granzyme B
IFN-α
IFN-γ
IL-1β
IL-1RA
IL-2
IL-4
IL-6
IL-8
IL-10
IL-12 (p70)
IL-12/23 (p40)
IL-15
IL-17A
IL-18
MCP-1
MIP-1β
TNF-α

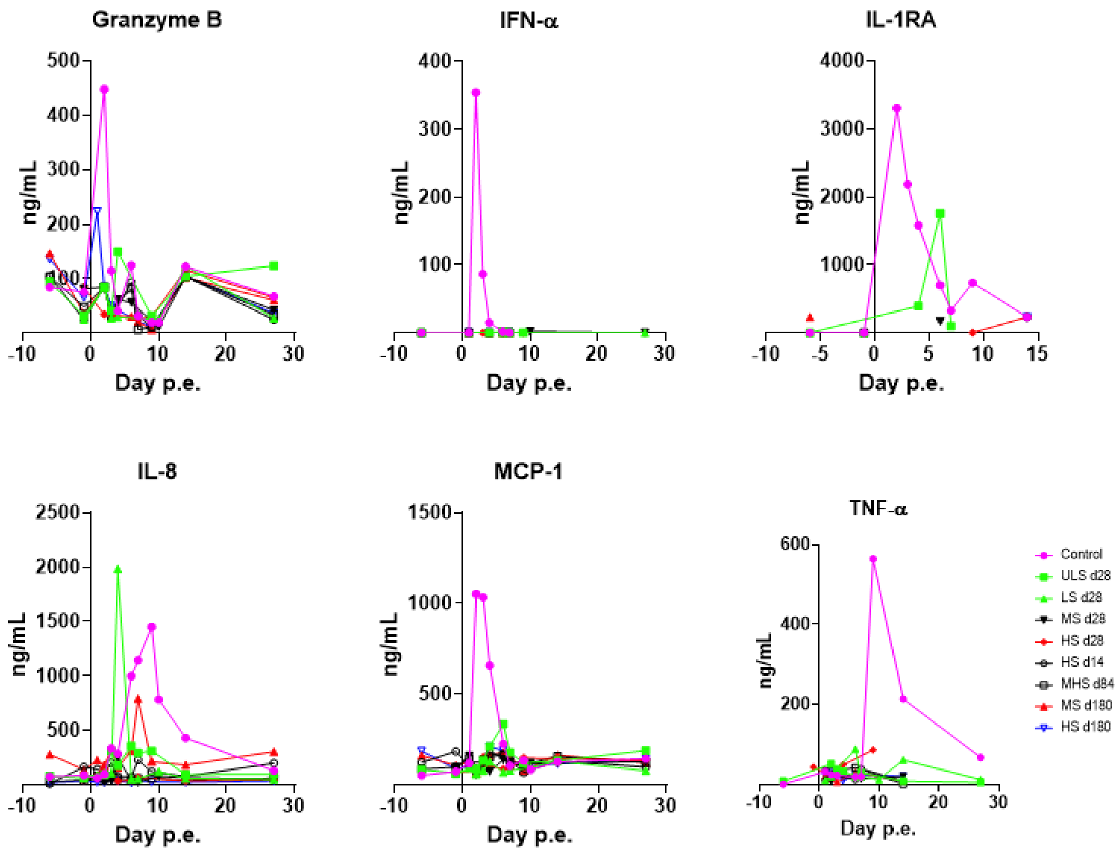

Figure S 3: Expression of inflammatory markers. Comparison of inflammatory response in VLA1553 Phase 1 and non-immune serum treated NHPs. IFN-γ, IL-2, IL-4, IL-6, IL-8, IL-10, IL-12/IL-13 (p40), IL-18 and TNF-α were measured using the Milliplex system and Granzyme B, IFN-α, IL-1β, IL-1RA, IL-12 (p70), IL-15, IL-17A, MCP-1 and MIP-1β were measured using the ProcartaPlex system. p.e.: post-exposure.

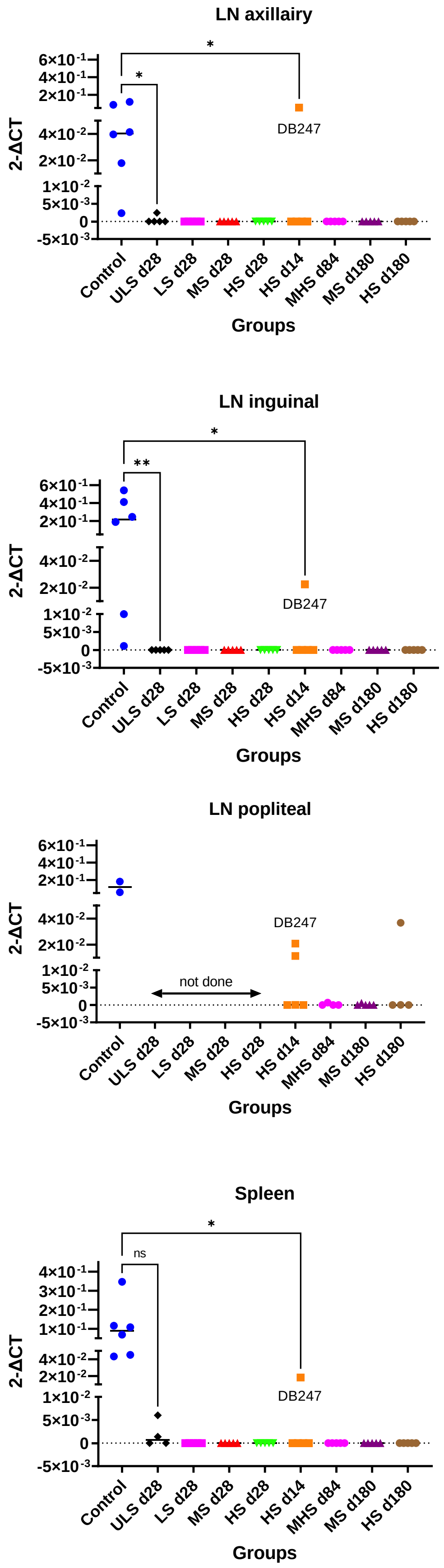
LN axillairy
2-ΔCT
DB247
*
Groups
Control
ULS d28
LS d28
MS d28
HS d28
HS d14
MHS d84
MS d180
HS d180
LN inguinal
2-ΔCT
DB247
**
*
Groups
LN popliteal
2-ΔCT
not done
DB247
Groups
Spleen
2-ΔCT
ns
*
DB247
Groups

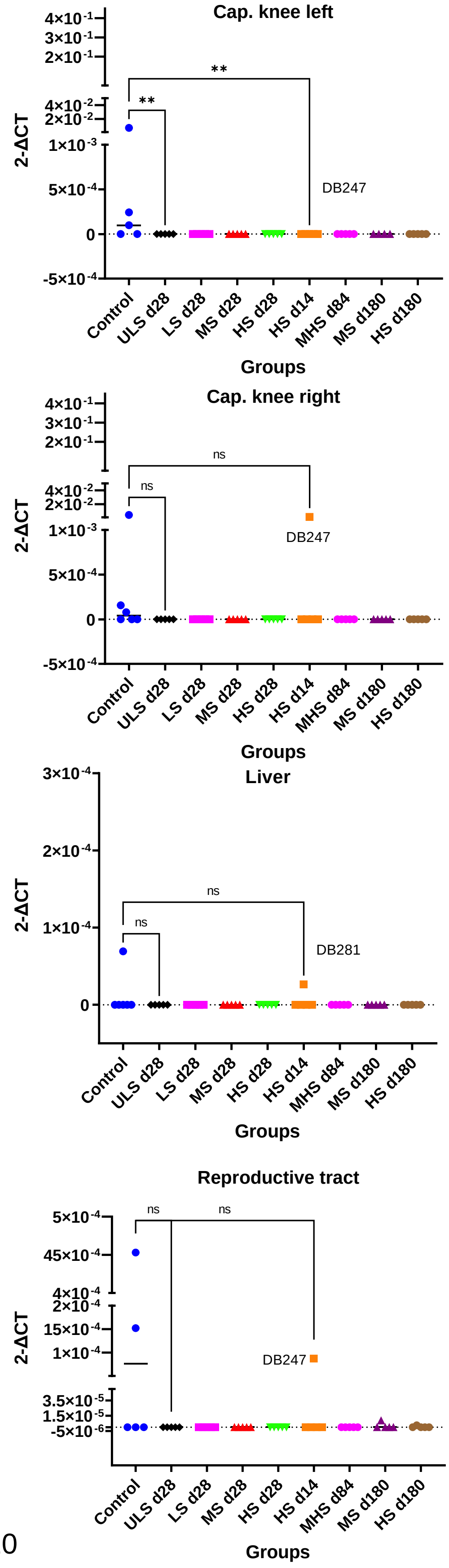
Cap. knee left
2-ΔCT
**
DB247
Groups
Control
ULS d28
LS d28
MS d28
HS d28
HS d14
MHS d84
MS d180
HS d180
Cap. knee right
2-ΔCT
ns
DB247
Groups
Liver
2-ΔCT
ns
DB281
Groups
Reproductive tract
2-ΔCT
ns
DB247
Groups

Figure S 4: CHIKV RNA titers in tissues day 28 post challenge. Results are expressed as the ratio CHIKV RNA / GAPDH RNA = 2-ΔCT. The median is plotted. Human non-immune sera injected animals are shown in blue; animals receiving the LS d28 shown in square pink; animals receiving MS d28 shown in red; animals receiving ULS d28 shown in black; animals receiving HS d28 shown in bright green; animals receiving HS d14 shown in orange; animals receiving day 84 medium high titer sera shown in round pink (MHS d84); animals receiving MS d180 are shown in purple and animals receiving HS d180 are shown in brown. ANOVA non parametric Kruskal-Wallis test $p<0.0001$; post test Dunn's multiple comparisons: * $p<0.05$; ** $p<0.01$, *** $p<0.001$. To note, for reproductive tract, results are combined values from vagina of the female and seminal vesicle from the male. Below limit of quantification (CHIKV Cq ≥35), below limit of detection (1E-07). LN: lymph node; cap.: capsule

Table S 3: Sera from Yoon *et al*., 2015 tested in µPRNT compared to $PRNT_{50}$ or $PRNT_{80}$ titers reported by AFRIMS.

Sample ID	$\mu PRNT_{50}$ Valneva	$PRNT_{80}$ Titer AFRIMS	Ratio $\mu PRNT_{50}/PRNT_{80}$
CPC C 0004 00 Y01 S01 B 001	<20	<10	-
CPC C 0004 00 Y02 S02 B 002	299	64	4.67
CPC C 0047 00 Y01 S01 B 001	<20	<10	-
CPC C 0047 00 Y02 S02 B 002	2086	537	3.88
CPC C 0092 00 Y01 S01 B 001	<20	<10	-
CPC C 0092 00 Y02 S02 B 002	1075	415	2.59
CPC C 0097 00 Y01 S01 B 001	<20	<10	-
CPC C 0097 00 Y02 S02 B 002	2621	661	3.97
CPC C 0139 00 Y01 S01 B 001	<20	<10	-
CPC C 0139 00 Y02 S02 B 002	1216	408	2.98
CPC C 0147 00 Y01 S01 B 001	<20	<10	-
CPC C 0147 00 Y02 S02 B 002	1208	425	2.84
CPC C 0175 00 Y01 S01 B 001	<20	<10	-
CPC C 0175 00 Y02 S02 B 002	576	111	5.19
CPC C 0178 00 Y01 S01 B 001	<20	<10	-
CPC C 0178 00 Y02 S02 B 002	2417	625	3.87
CPC C 0273 00 Y01 S01 B 001	<20	<10	-
CPC C 0273 00 Y02 S02 B 002	2143	811	2.64
CPC C 0406 00 Y01 S01 B 001	<20	<10	-
CPC C 0406 00 Y02 S02 B 002	5297	569	9.31
CPC C 0458 00 Y01 S01 B 001	<20	<10	-
CPC C 0458 00 Y02 S02 B 002	804	238	3.38
CPC C 0658 00 Y01 S01 B 001	<20	<10	-
CPC C 0658 00 Y02 S02 B 002	1808	488	3.70
CPC C 0673 00 Y01 S01 B 001	<20	<10	-
CPC C 0673 00 Y02 S02 B 002	3105	3347	0.93

Sample ID	μ$PRNT_{50}$ Valneva	$PRNT_{80}$ Titer AFRIMS	Ratio μ$PRNT_{50}$/$PRNT_{80}$
CPC C 0751 00 Y01 S01 B 001	<20	<10	-
CPC C 0751 00 Y02 S02 B 002	289	122	2.37
CPC C 0757 00 Y01 S01 B 001	<20	<10	-
CPC C 0757 00 Y02 S02 B 002	1199	337	3.56
CPC C 0857 00 Y01 S01 B 001	<20	<10	-
CPC C 0857 00 Y02 S02 B 002	2824	1088	2.60
CPC C 0879 00 Y01 S01 B 001	<20	<10	-
CPC C 0879 00 Y02 S02 B 002	2904	347	8.37
CPC C 0901 00 Y01 S01 B 001	<20	<10	-
CPC C 0901 00 Y02 S02 B 002	355	86	4.13
CPC C 0043 00 Y01 S01 B 001	1019	284	3.59
CPC C 0043 00 Y02 S02 B 002	1514	390	3.88
CPC C 0114 00 Y01 S01 B 001	1266	705	1.80
CPC C 0114 00 Y02 S02 B 002	1875	717	2.61
CPC C 0190 00 Y01 S01 B 001	299	158	1.89
CPC C 0190 00 Y02 S02 B 002	170	203	0.84
CPC C 0278 00 Y01 S01 B 001	<20	<10	-
CPC C 0278 00 Y02 S02 B 002	2454	1228	2.00
CPC C 0421 00 Y01 S01 B 001	694	97	7.15
CPC C 0421 00 Y02 S02 B 002	349	147	2.38
CPC C 0567 00 Y01 S01 B 001	<20	<10	-
CPC C 0567 00 Y02 S02 B 002	942	116	8.12
CPC C 0607 00 Y01 S01 B 001	<20	<10	-
CPC C 0607 00 Y02 S02 B 002	2971	420	7.07
CPC C 0653 00 Y01 S01 B 001	3135	225	13.93
CPC C 0653 00 Y02 S02 B 002	1409	290	4.86
CPC C 0688 00 Y01 S01 B 001	<20	<10	-
CPC C 0688 00 Y02 S02 B 002	4136	2372	1.74

Sample ID	$\mu PRNT_{50}$ Valneva	$PRNT_{80}$ Titer AFRIMS	Ratio $\mu PRNT_{50}/PRNT_{80}$
CPC C 0699 00 Y01 S01 B 001	<20	<10	-
CPC C 0699 00 Y02 S02 B 002	3903	346	11.28
CPC C 0740 00 Y01 S01 B 001	<20	<10	-
CPC C 0740 00 Y02 S02 B 002	2001	297	6.74
CPC C 0814 00 Y01 S01 B 001	<20	<10	-
CPC C 0814 00 Y02 S02 B 002	1745	544	3.21
CPC C 0834 00 Y01 S01 B 001	<20	<10	-
CPC C 0834 00 Y02 S02 B 002	5133	674	7.62
CPC C 0847 00 Y01 S01 B 001	<20	<10	-
CPC C 0847 00 Y02 S02 B 002	2263	602	3.76
CPC C 0950 00 Y01 S01 B 001	702	140	5.01
CPC C 0950 00 Y02 S02 B 002	810	236	3.43
		**GMR**	**3.73**
		**Lower 99% CI of geo. mean ratio**	**2.86**
		**Upper 99% CI of geo. mean ratio**	**4.87**

PRNT: plaque reduction neutralization test; $\mu PRNT_{50}$: neutralization titer determined in a microneutralization assay (96 well format) using a 50% plaque reduction; $PRNT_{80}$: neutralization titer using a 80% plaque reduction; CI: confidence interval; GMR: geometric mean ratio.
